# Increased Screen Use on Days With Increased Perceived COVID-19-Related Confinements—A Day Level Ecological Momentary Assessment Study

**DOI:** 10.3389/fpubh.2020.623205

**Published:** 2021-02-02

**Authors:** Ann-Kathrin Arend, Jens Blechert, Björn Pannicke, Julia Reichenberger

**Affiliations:** Department of Psychology, Centre for Cognitive Neuroscience, Paris-Lodron-University of Salzburg, Salzburg, Austria

**Keywords:** COVID-19, confinements, screen use, day structure, ecological momentary assessment (EMA)

## Abstract

**Introduction:** Coronavirus 2019 (COVID-19) quickly evolved into a global pandemic in early 2020, and most countries enforced social confinements to reduce transmission. This seems to dovetail with increasing, potentially problematic, screen use habits, such as gaming and “binge-watching.” Yet, the subjective experience of the common confinements may vary not only between individuals depending on age, sex, and living conditions (i.e., living alone) but also within individuals from day to day: confinements might interfere with habitual activity schedules more strongly on some days than on others. Such dynamic confinement experience has not been studied in relation to screen use yet but might guide targeted intervention.

**Method:** In total, 102 participants (*n* = 83 female, *n* = 80 university students) completed 14 days of ecological momentary assessment during a COVID-19-related lockdown in Germany and Austria. Each evening, they indicated the extent to which they felt restricted by confinements in their social and work lives and whether they engaged in unusually high and intense levels of television watching, social media use, news consumption, internet surfing, and gaming. They also reported on how much they experienced their day to be structured.

**Results:** Experienced work confinements were positively associated with social media usage. Further, work confinements were positively associated with gaming in males and with news consumption, especially in individuals living alone. Social confinements were positively associated with watching television especially in younger participants and with social media consumption in younger participants. Higher experienced day structure was related to less television watching, gaming, and internet surfing but more news consumption.

**Discussion:** Screen use behaviors increased with higher confinements within person, dependent on sex, age, and living situation. Such knowledge allows tailoring on the person level (who should be addressed?) and the time level (when should interventions be scheduled?) as the negative consequences of excessive screen use behaviors on mental and physical health are well-documented. One potential low-threshold intervention might be day-structuring.

## Introduction

Throughout 2020, the coronavirus 2019 (COVID-19) evolved into a global pandemic with a negative impact on physical and psychological health [e.g., increased anxiety and depression; (1)]. To slow down the spreading of the coronavirus and to stabilize overstrained healthcare systems, most countries enforced partial lockdowns and confinements on social interaction. However, these lockdowns were associated with higher levels of stress, anxiety, and depression especially in younger individuals (2).

Besides having to deal with the uncertainty about the possible consequences of COVID-19 infections, such as medical complications, citizens were faced with novel situations as they experienced a loss of their usual routine and had to adapt to social and work confinements (e.g., reduced social contact, home-office, and -schooling). This might cause boredom, frustration, and feelings of isolation (3). Additionally, the reduction of recreational outdoor activities and social contact limits the available sources for habitually used positive reinforcement and thus protective factors of psychological health (4–6). To sum, these deprivations in concert with the breakdown of daily routines experienced during COVID-19-related confinements have the potential to increase reinforcing (indoor) behaviors that are still accessible and easily available. To illustrate, it has previously been shown that potentially problematic behaviors, such as increased consumption of food (7, 8), alcohol (9), or cannabis (10), become more likely during COVID-19-related confinements.

Another potential source of easily accessible and highly reinforcing activities, especially in highly technologized societies, may be intense screen use behaviors, such as watching television, gaming, internet surfing, or social media usage (11, 12). To exemplify, studies showed that the overall screen time increased during lockdown in children and adolescents (13, 14), as well as in office workers and students (15–19). More specifically, recent studies reported increased screen use habits, such as gaming (20), watching television, or even binge-watching (7, 21, 22), as well as social media use [e.g., (21, 23, 24)], during COVID-19-related confinements (8).

Such excessive screen use behaviors can be associated with negative effects on psychological well-being during COVID-19-related confinements: students were negatively affected in their sleep quality, sleep duration, physical well-being, and mental health by excessive screen time (15); increased social media use was associated with a greater tendency to be diagnosed with depression or anxiety (24); finally, more time consuming news led to higher levels of anxiety and stress (3, 25). Moreover, individuals during adolescence and young adulthood may be especially vulnerable to develop excessive, impulsive–compulsive screen use behaviors corresponding to the concept of “behavioral addiction” (11, 26). Hence, examining screen use behaviors during COVID-19 confinements seems important to prevent the negative health outcomes mentioned above.

The COVID-19 situation and related confinements have been very dynamic with new regulations introduced almost on a daily basis. Moreover, within individuals, the *subjective* experience of these *objective* confinements might have varied significantly from day to day: individuals may have experienced confinements as more impacting on days where they used to engage in activities that are now restricted (e.g., outdoor recreation, social gatherings on weekends, or work meetings on work days). Similarly, the confinements might have affected different life areas (e.g., individuals may experience more work-related confinements during the week but suffer more from social confinements on the weekend, depending on their usual routines). This creates much variability within individuals (i.e., day-to-day variability in experienced confinements). As some new findings showed, such situational factors [i.e., varying degrees of experienced confinements; (18)] may contribute to increased screen use. Hence, it may be most appropriate to assess various perceived COVID-19-related confinements and screen use on a daily basis using ecological momentary assessment (EMA) that accounts for this within-person variance.

Additionally, between individuals, confinements may have different effects depending on different professions or living situations (e.g., some individuals stopped working or lost their job, whereas others had to work in home-office). Thus, also between-person variables need to be acknowledged: demographic and environmental factors, i.e., age, sex, or living alone, have been linked to excessive screen use [i.e., online gamers typically are male, young, university graduates, and live alone; (27)].

It has been shown that the *subjective* experience of social isolation is as likely to predict negative effects on well-being, compared with *objective* social isolation (28). Thus, we focused on the subjective experienced degree of social and work confinements from day to day and their association with increases of screen use behaviors, but additionally assessed whether the participants lived alone or together with others, as individuals who lived alone might have been objectively more isolated during the lockdown period.

On that background, the present naturalistic study examined the relationship of daily varying experiences of COVID-19-related confinements with screen use behaviors, as well as the moderating roles of demographic, environmental, and situational factors, in this relationship across 14 days of day level EMA. Based on the literature reviewed above, it was hypothesized that increased subjective work and social confinements would be associated with an increased probability for screen use behaviors (television watching, social media usage, internet surfing, gaming, and news consumption) within person. Additionally, we hypothesized that age, sex, and living situation (alone or with others) may moderate the increase of different screen use behaviors with regard to the experience of increased confinements. As it was recommended that a more structured daily routine should be followed to avoid excessive engagement in screen use behaviors (12), we hypothesized that days marked by a more structured daily routine would be negatively associated with the probability for increased engagement in screen use behaviors on that day.

## Materials and Methods

### Participants

In total, 102 participants (*n* = 83 female) were included. The participants were recruited via social media postings and mailing lists in Germany and Austria. All participants completed the study during a COVID-19-related lockdown (from March to May 2020) in both countries. Participants were recruited for an EMA study on the influence of COVID-19-related confinements on eating behavior. The sample mainly consisted of university students (78.4%), followed by employees (15.7%) and few self-employed, homemakers, pupils, and retirees (5.9%). Thus, mainly young adults participated in the study (age: *M* = 25.5, *SD* = 9.20, range 18–71 years; 25th percentile 20.0, 75th percentile 26.3 years). Most participants reported to live with others (*n* = 86), whereas the rest lived alone (*n* = 16). The ethics committee of the University of Salzburg approved the study, and all participants signed an informed consent form approved by the ethics committee of the University of Salzburg.

### Measures

#### Sociodemographic Measures

Participants reported sociodemographic data via an online questionnaire (i.e., sex, age, whether they live alone or together with others, and other unrelated measures).

#### EMA Measures

Participants completed five EMA signals a day, repeatedly asking about emotions, stress, eating behavior, and other variables that are not of interest for the present study. At the last signal of each day, participants indicated the extent to which they felt restricted by confinements in their (a) social and (b) work lives via visual analog scales in the form of continuous rating sliders (“How much did you feel confined in your social life today?” and “How much did you feel confined in your work life today?”: from *not at all* [0] to *very much* [100]). Further, they reported whether they engaged, more than they usually do, in one or several of five screen use behaviors (“Did you engage in one or more of the following activities in your leisure time today? In comparison with usual intensified and increased.” television watching, social media use, news consumption, internet surfing, and gaming: *yes* or *no* for each screen use behavior). Finally, the participants reported how much they experienced their day to be structured (“How structured was your day?”: from *not at all* [0] to *very much* [100]).

### Procedure

At the start of the study, all participants signed the informed consent form and completed an online questionnaire asking about the abovementioned sociodemographic factors. Via phone, participants were instructed on how to install and use the customized EMA application *PsyDiary*. The duration of the EMA protocol lasted for 14 days, with five signal-contingent prompts per day^1^. At the last prompt of each day (at 09:00 pm), participants answered the questions regarding confinements in social and work lives, engagement in unusually high and intense levels of screen use behaviors, and the experienced day structure. In general, participants could delay the signal response for up to 1 h while later responses were treated as missing. All participants were compensated for their participation with 3–5 course credits (depending on their EMA compliance) and a personalized feedback of their data.

### Statistical Analyses

A multilevel modeling (MLM) approach was used to account for nesting of within-person (prompts, level 1, e.g., day-to-day variation in experienced confinement) and between-person (individuals, level 2, e.g., sociodemographic data) variance. Thus, social and work confinements, as well as screen use behaviors, were modeled as level 1 variables, whereas sex, age, and living situation (i.e., living alone or together with others) were modeled as level 2 variables. To account for the binomial distribution of the outcome measures (i.e., reports of increased television watching, social media use, news consumption, gaming, and internet surfing), the Bernoulli–MLM models were used. Level 1 variables (social and work confinements and day structure) were person-mean centered (centered within person), and continuous level 2 variables (age) were grand-mean centered (centered around the group mean).

In a first step, MLM Null-models (including only a random intercept for participants) were tested for significance. Significance of these tests indicated a nested data structure. Thus, MLMs with random effect structure were preferable to general linear regression models. Therefore, random intercepts and random slopes for each participant were added to the fixed factors (work and social confinements), to model variance between and within individuals.

To account for the expected moderating roles of sex, age, and living situation on certain screen use behaviors, additional interaction models were conducted. To exemplify, increased gaming was modeled as a dependent variable with COVID-19-related confinements, and sex and their interaction as independent variables. For all outcomes, additional models with multilevel interactions separately including sex, age and living situation were calculated. All MLMs were setup with nested random effect structure (30) and analyzed in HLM7 (31).

Additionally, we used Rstudio (32) and the packages lme4 (33) and nlme (34) to recalculate our models and test whether all assumptions of MLMs were met for our data. Linearity of the data was checked upon visual inspection by plotting the residuals of each model vs. the observed outcome values. Homogeneity of residual variance was checked by a variation of the Levene's test: the residual variance from each participant was extracted, and an ANOVA of the between subject residuals was calculated (for each model). Normal distribution of residuals was checked upon visual inspection of Q–Q plots of the random effects of each model.

Data from *n* = 3 participants were excluded from the analyses due to insufficient data quantity, as they answered <50% of all EMA prompts.

## Results

### Descriptive Data

In total, 1,257 EMA evening prompts were answered and used for analyses. On average, 12.2 out of 14 EMA prompts per participant were answered, equaling to a good compliance of 87.4% (*SD* = 13.1, range 50–100%). On average, participants reported mild to moderate confinements in their social (*M* = 33.2, *SD* = 28.1, range 0–100) and work lives (*M* = 24.4, *SD* = 30.0, range 0–100) and moderately structured days (*M* = 49.7, *SD* = 27.5, range 0–100). Increased screen use behaviors compared with usual were reported, as can be seen in Table 1.

**Table 1:** Amount of increased and intensified screen use behaviors compared with usual.

**Increased screen use behavior**	**Percentage of all observations Yes, more than usual–no, as/less than usual**		***n*** **Yes–no**	
TV	40.7%	49.3%	511	746
Social media	43.8%	46.2%	550	707
News^3^	29.7%	70.3%	373	884
Internet	23.9%	76.1%	301	956
Gaming^4^	9.07%	90.9%	114	1,143

*Frequency of prompts (out of the 1,257 prompts answered by all participants) with increased screen use behaviors compared with usual. Assessed in the evening (09:00 pm−10:00 pm) on 14 consecutive days*.

3 *Homogeneity of variance for the percentage of increased news consumption of all answered EMA prompts was not given between participants who lived alone compared with those who lived with others. Still, participants who lived alone did not significantly differ from participants who lived with others, in their reports of increased news consumption [t_(27)_ = −1.87, SE = 6.74, p = 0.072]*.

4 *Homogeneity of variance for the percentage of increased gaming of all answered EMA prompts was not given between men and women. Men reported increased gaming for a significantly higher percentage of their answers (M = 30.1%, SD = 36.5%) than women (M = 4.4%, SD = 14.1%) [t_(19)_ = −3.02, SE = 8.52, p = 0.007]. Only 17.4% (n = 15) of the women, but 81.3% (n = 13) of the men reported increased gaming at least once throughout the 14 days of EMA*.

### EMA Measures

#### Effects of Work Confinements^2^

**Figure 1:**
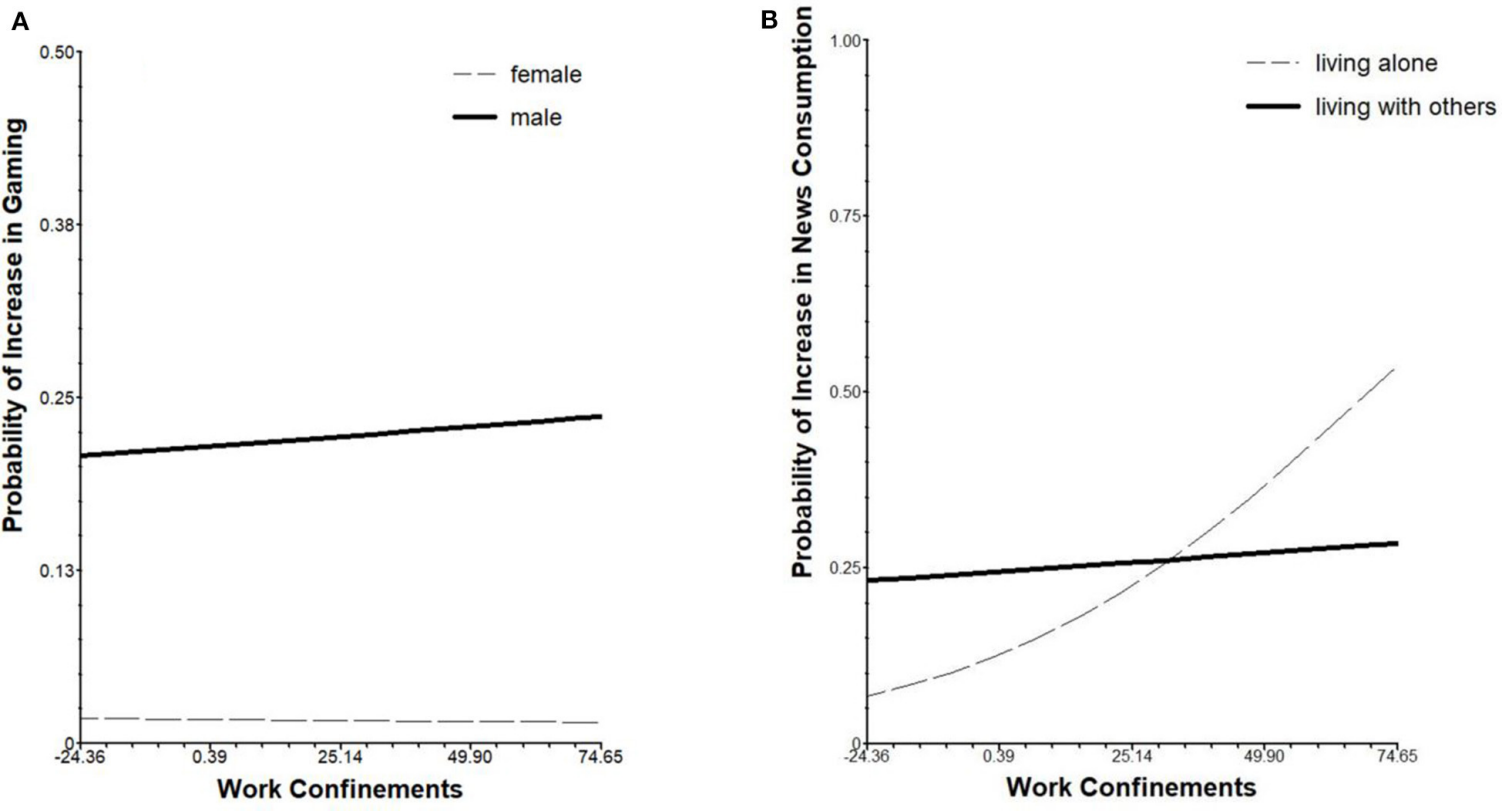
From left to right: **(A)** simple slopes of the probability for increased gaming in relation to experience of work confinements moderated by sex, **(B)** simple slopes of the probability for increased news consumption in relation to experience of work confinements moderated by living situation. Scaling of the x-axis is based on the entire range of person-mean centered scores of work confinements.

Higher experienced work confinements were positively associated with a higher probability of social media usage within participants (β_01_ = 0.006, *SE* = 0.002, *p* = 0.004). An association of work confinements and reports of increased gaming (β_01_ = −0.003, *SE* = 0.001, *p* = 0.024) was moderated by sex, yielding a positive association of work confinements and increased gaming only in males (β_11_ = 0.006, *SE* = 0.002, *p* = 0.006), as can be seen in Figure 1A. Further, work confinements were positively associated with reports of news consumption, especially in participants who lived alone (β_11_ = −0.015, *SE* = 0.006, *p* = 0.009), as can be seen in Figure 1B. No further associations of work confinements and increases in other screen use behaviors were found, and no further interactions of work confinements and age, sex, or living situation were found regarding the different dependent variables of increased screen use behaviors.

#### Effects of Social Confinements^2^

**Figure 2:**
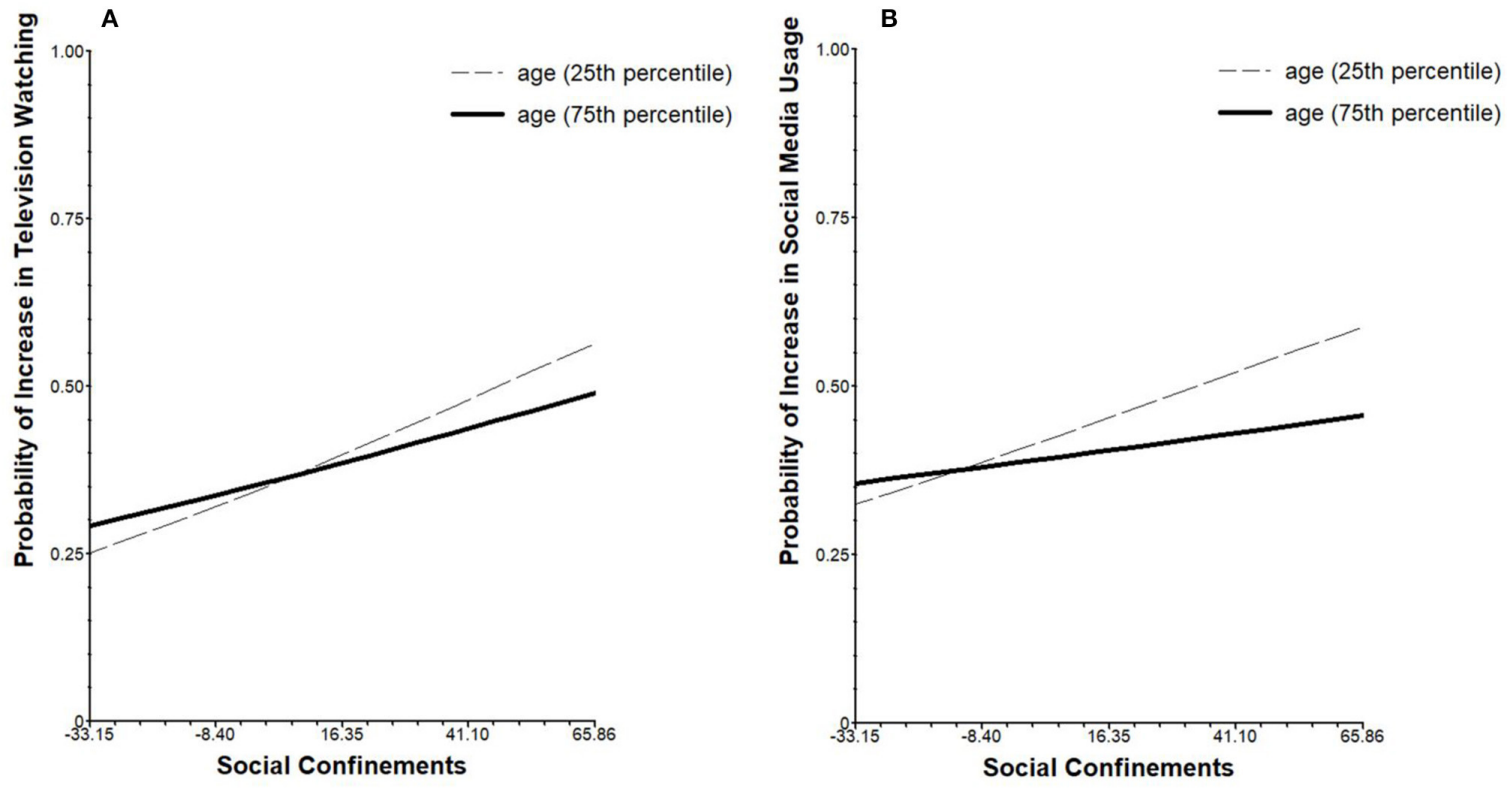
From left to right: **(A)** simple slopes of the probability for increased television watching in relation to experience of social confinements moderated by age, **(B)** simple slopes of the probability for increased social media usage in relation to experience of social confinements moderated by age. For visualization only, the 25th percentile (20.0 years) and 75th percentile (26.3 years) of the participants are displayed to highlight the interaction of age and social confinements. Scaling of the x-axis is based on the entire range of person-mean centered scores of social confinements.

Higher social confinements were positively associated with higher probability of reports of increased television watching within participants (β_01_ = 0.007, *SE* = 0.003, *p* = 0.019). This association was moderated by age so that it was stronger in younger participants (β_11_ = −0.001, *SE* = 0.0002, *p* = 0.004), as can be seen in Figure 2A. Social confinements were also significantly associated with higher probability of reports of increased social media consumption in younger than in older participants (β_11_ = −0.001, *SE* = 0.0002, *p* = 0.001), as can be seen in Figure 2B. No further associations of social confinements and increases in other screen use behaviors were found, and no further interactions of social confinements and age, sex, or living situation were found regarding the different dependent variables of increased screen use behaviors.

All reported significant main effects of social and work confinements on increased screen use behaviors remained significant after combining social and work confinements into one model to control for shared variance. For detailed tables of all models and results, see Supplementary Materials.

#### Effects of Day Structure^2^

A higher experienced day structure was related to a lower probability of reports of increased television watching (β_01_ = −0.012, *SE* = 0.003, *p* < 0.001), gaming (β_01_ = −0.005, *SE* = 0.002, *p* = 0.014), and internet surfing (β_01_ = −0.006, *SE* = 0.002, *p* = 0.008) within participants, but to a higher probability news consumption within participants (β_01_ = 0.005, *SE* = 0.002, *p* = 0.049).

## Discussion

The present study examined the impact of subjectively perceived work and social COVID-19-related confinements on the increase of different screen use behaviors in daily life using day level EMA across 14 days. Due to the highly dynamic COVID-19 situation and the fact that confinements might interfere with habitual activity schedules more strongly on some days than on others, we were explicitly interested in within-person relationships of these variables, but also examined the role of potential moderators of these associations, such as age, sex, and living alone vs. with others.

Results showed that participants reported increased screen use during leisure time, mostly social media and television watching, followed by news consumption, other internet usage, and gaming. In line with previous research, screen use increased during COVID-19-related confinements compared with usual every day conditions [e.g., (13–17, 19)]. Potentially, the low prevalence of increased gaming in our study is due to our mostly female sample, as previous research showed excessive gaming being prevalent mostly in male individuals (27). Indeed, increased gaming was reported by most male individuals in our sample but only few female participants, so that future studies might profit from a sample with a higher percentage of male individuals.

Increased screen use during COVID-19 may have positive and negative side effects. On the one hand, increased screen use may aid individuals in coping with the COVID-19 crisis. On the other hand, it may worsen psychological well-being. To illustrate, increased social media use might enable individuals during the COVID-19-related confinement to stay in contact with others and overcome social distancing (23), and increased news consumption may help individuals to stay informed and cope with COVID-19-related uncertainty (35, 36). Still, excessive screen use is related to decrease physiological and psychological well-being, and increased news consumption may even be related to greater fear about infection.

Moreover, our results showed that the effects of COVID-19-related confinements differ within individuals on a day-to-day basis as the subjectively experienced degree and the life domain of confinements vary: work confinements were positively associated with the probability of increased social media usage, whereas social confinements were positively associated with the probability of increased television watching within participants. This suggests that COVID-19-related confinements may not be seen as temporally stable or as an “all or nothing” factor, but significant day-to-day variations exist and those go along with variations in screen use. To exemplify, within-person confinements might interfere with personal recreational habits on one-day (i.e., leading to increased television watching) and with important job tasks on another day (i.e., leading to increased social media usage), yet on another day, the confinements may not interfere with any activities or duties at all (e.g., weekend day at home with family). Hence, instead of focusing on confinements as a dichotomous state (confined, non-confined), a more fine-grained assessment may be more appropriate for explaining screen use behavior and for intervening on it in case of problematic levels.

Regarding interventions, one potential protective factor might be a well-structured day, which has already been recommended by previous research (12): our data showed that the degree to which participants experienced their day to be structured was negatively associated with increased screen use behaviors (television watching, gaming, and internet surfing) within participants. Only news consumption was positively associated with the degree of day structure, but news consumption might inherently structure the day. Thus, the present study calls for targeted prevention and intervention and sheds some light on a potential low-threshold intervention in the form of day-structuring and planning. Such interventions might aid individuals in managing excessive screen use behaviors by preplanning different duties and recreational activities beforehand to minimize the degree of confinements actually experienced later on, due to a lack of preparation.

Further, our results provided new insights by showing who is more affected in their screen use behaviors by COVID-19-related confinements than others are: increased gaming was reported by males more on days with more work confinements. Increased news consumption was seen especially in individuals who live alone on days with more work confinements. Additionally, increased television watching and social media usage were reported by younger participants on days with more social confinements. Thus, to some degree, our results underpin previous studies [e.g., (27), (37)] showing that young, male, and individuals who live alone, may be most vulnerable for certain excessive screen use behaviors and thus represent an important target population for prevention and intervention strategies.

Increased screen use behaviors may further be problematic, as subgroups of especially vulnerable individuals may be at risk of developing chronic and excessive usage patterns. Previous research showed that such behaviors relate to poorer psychological and physical well-being (3, 15, 24, 25). Additionally, these behaviors may become addictive over time, so that several researchers argued that addiction-related disorders need special attention during the COVID-19 pandemic (38). Such mostly sedentary behaviors additionally seem to constitute a risk factor for weight gain during the COVID-19 pandemic (39). These points should be considered in prevention and intervention approaches in order to help individuals adapt their health behaviors. Several guidelines have been developed recently, providing advices on how to manage excessive behaviors (i.e., screen use) during the COVID-19 pandemic [e.g., (12, 40)] and to prevent and treat addictive behavior-related disorders (38). Still, the current study calls for targeted preventions and interventions toward particularly vulnerable individuals (i.e., between-person relationships regarding sex, age, and living alone).

### Limitations and Future Research

The study mainly sampled university students at younger age who are at risk for developing chronically excessive behaviors, such as internet addiction (37), and experiencing a decline in psychological well-being during the COVID-19 pandemic, even more so in female university students (41). Nevertheless, this limits the generalizability of the present findings and calls for a replication in a sample with more diverse socio-economic characteristics. Additionally, the current study only covered 14 days of EMA assessment, mainly to limit participant burden and enhance compliance. However, long-term trajectories may be interesting, particularly with regard to the potential chronicity of addictive-like behaviors.

### Clinical Impact and Future Directions of Research

In case of prolonged COVID-19-related confinements (i.e., potential upcoming lockdowns), a direct application of our findings would be to tailor a day-structuring intervention to counteract excessive screen use behaviors in vulnerable individuals. The possible intervention techniques would be planning of recreational activities, for example, through implementation intentions (42), situational specific action plans with an if-then structure. According to our data, especially young adults might profit from such interventions to limit increased television watching, whereas males may limit increased gaming.

Especially children and adolescents showed excessive screen use behaviors during COVID-19-related confinements [i.e., (13, 43–45)]; future research could examine if day-structuring might also be preventative in these subgroups. Simultaneously, a day-structuring intervention might even be useful to reduce other potentially problematic behaviors during COVID-19-related confinements, but this remains to be examined in future research. Moreover, day-structuring might also aid in deliberate integration and realization of healthy recreational activities, which might add to the overall psychological and physiological well-being during COVID-19-related confinements.

Apart from day-structuring, cognitive interventions might also be useful: results of the current study suggest that the subjective experience of COVID-19-related confinements seems especially important when it comes to increased screen use behaviors. Previous research makes it seem likely that the subjective experience of quarantine as either enforced vs. voluntary resembles an important differentiation with regard to health outcomes (3). Hence, applying framing of confinement measures as appeals to each and everyone's responsibility for the community (e.g., to avoid transmission, to protect the beloved ones), and emphasizing some positive aspects (e.g., time for family, time to recover from work stress) might also aid in avoiding increased screen use behaviors. As a result, future research should build on these findings and develop targeted and temporally precise interventions to tackle the negative psychological outcomes of COVID-19-related confinements.

## Data Availability Statement

The raw data supporting the conclusions of this article will be made available by the authors, without undue reservation.

## Ethics Statement

The studies involving human participants were reviewed and approved by Ethics committee of the University of Salzburg. The patients/participants provided their written informed consent to participate in this study.

## Author Contributions

A-KA, JB, BP, and JR were involved in designing the present study. A-KA, BP, and JR contributed to the data acquisition and preparation. A-KA conducted the data analyses and wrote the first draft of the present manuscript. JB and JR revised the present manuscript. All authors approved the submitted version.

## Conflict of Interest

The authors declare that the research was conducted in the absence of any commercial or financial relationships that could be construed as a potential conflict of interest.

## Footnotes

^1^The assessments of the other prompts were focused on eating behavior. The data are reported elsewhere (29).

^2^For all reported models, the assumptions of linearity (upon visual inspection of plots), homogeneity of residual variance (all *p*'s > 0.500), and normal distribution of residuals (upon visual inspection of plots) were met.
