# Supplementary material for: Increased Screen Use on Days With Increased Perceived COVID-19-Related Confinements—A Day Level Ecological Momentary Assessment Study
Source: Front Public Health. 2021 Feb 2;8:623205. doi: 10.3389/fpubh.2020.623205 (PMC7902048; doi:10.3389/fpubh.2020.623205)
Supplement: Supplementary file 1 [file Data_Sheet_1.docx]

**Supplementary Materials**

**Supplementary Tables**

Table S1. Increased screen use behaviors during lockdown predicted by work confinement.

| Outcome | Coefficients β (*SE*) | *p-*value |
| --- | --- | --- |
| TV | 0.002 (0.003) | .480 |
| Social Media | ***0.006 (0.002)*** | ***.004*** |
| News | *0.004 (0.002)* | *.079* |
| Gaming | **-0.003 (0.001)** | **.024** |
| Internet | -0.001 (0.003) | .582 |
| *Note.* Italic beta coefficients and *p*-values are significant at *p* < .1. Bold beta coefficients and *p*-values are significant at *p* < .05. Bold and italic beta coefficients and *p*-values are significant at *p* < .01. | | |

Table S2. Increased screen use behaviors during lockdown predicted by work confinement and controlled for sex.

| Predictor | Coefficients β (*SE*) | *p-*value |
| --- | --- | --- |
| Outcome: TV |  |  |
| Intercept | ***-0.542 (0.142)*** | ***< .001*** |
| Work confinement | 0.002 (0.002) | .443 |
| Sex | *0.564 (0.326)* | *.086* |
| Outcome: Social Media |  |  |
| Intercept | *-0.288 (0.157)* | *.069* |
| Work confinement | **0.005 (0.002)** | **.029** |
| Sex | -0.053 (0.409) | .898 |
| Outcome: News |  |  |
| Intercept | ***-0.940 (0.151)*** | ***< .001*** |
| Work confinement | *0.005 (0.002)* | *.055* |
| Sex | 0.339 (0.310) | .277 |
| Outcome: Gaming |  |  |
| Intercept | ***-2.586 (0.123)*** | ***< .001*** |
| Work confinement | ***-0.004 (0.001)*** | ***< .001*** |
| Sex | ***1.792 (0.370)*** | ***< .001*** |
| Outcome: Internet |  |  |
| Intercept | ***-1.324 (0.148)*** | ***< .001*** |
| Work confinement | 0.001 (0.003) | .678 |
| Sex | **0.707 (0.353)** | **.048** |
| *Note.* Italic beta coefficients and *p*-values are significant at *p* < .1. Bold beta coefficients and *p*-values are significant at *p* < .05. Bold and italic beta coefficients and *p*-values are significant at *p* < .01. | | |

Table S3. Increased screen use behaviors during lockdown predicted by work confinement and controlled for age.

| Predictor | Coefficients β (*SE*) | *p-*value |
| --- | --- | --- |
| Outcome: TV |  |  |
| Intercept | ***-0.434 (0.129)*** | ***.001*** |
| Work confinement | 0.002 (0.003) | .491 |
| Age | 0.007 (0.017) | .673 |
| Outcome: Social Media |  |  |
| Intercept | **-0.298 (0.145)** | **.043** |
| Work confinement | **0.005 (0.002)** | **.028** |
| Age | -0.007 (0.013) | .598 |
| Outcome: News |  |  |
| Intercept | ***-0.891 (0.130)*** | ***< .001*** |
| Work confinement | *0.005 (0.002)* | *.054* |
| Age | **0.037 (0.015)** | **.017** |
| Outcome: Gaming |  |  |
| Intercept | ***-2.046 (0.131)*** | ***< .001*** |
| Work confinement | **-0.003 (0.001)** | **.014** |
| Age | *-0.013 (0.007)* | *.071* |
| Outcome: Internet |  |  |
| Intercept | ***-1.182 (0.136)*** | ***< .001*** |
| Work confinement | 0.001 (0.003) | .688 |
| Age | 0.017 (0.014) | .251 |
| *Note.* Italic beta coefficients and *p*-values are significant at *p* < .1. Bold beta coefficients and *p*-values are significant at *p* < .05. Bold and italic beta coefficients and *p*-values are significant at *p* < .01. | | |

Table S4. Increased screen use behaviors during lockdown predicted by work confinement and controlled for living situation (living alone or together with others).

| Predictor | Coefficients β (*SE*) | *p-*value |
| --- | --- | --- |
| Outcome: TV |  |  |
| Intercept | *-0.691 (0.374)* | *.067* |
| Work confinement | 0.002 (0.003) | .498 |
| Living situation | 0.304 (0.397) | .445 |
| Outcome: Social Media |  |  |
| Intercept | *-0.683 (0.344)* | *.050* |
| Work confinement | **0.005 (0.002)** | **.030** |
| Living situation | 0.456 (0.380) | .233 |
| Outcome: News |  |  |
| Intercept | ***-1.460 (0.278)*** | ***< .001*** |
| Work confinement | *0.005 (0.002)* | *.061* |
| Living situation | **0.685 (0.314)** | **.032** |
| Outcome: Gaming |  |  |
| Intercept | ***-1.854 (0.378)*** | ***< .001*** |
| Work confinement | **-0.003 (0.001)** | **.014** |
| Living situation | -0.225 (0.402) | .577 |
| Outcome: Internet |  |  |
| Intercept | ***-1.159 (0.288)*** | ***< .001*** |
| Work confinement | 0.001 (0.003) | .684 |
| Living situation | -0.021 (0.326) | .948 |
| *Note.* Italic beta coefficients and *p*-values are significant at *p* < .1. Bold beta coefficients and *p*-values are significant at *p* < .05. Bold and italic beta coefficients and *p*-values are significant at *p* < .01. | | |

Table S5. Interaction effect of work confinement and sex on increased gaming and living situation on increased news consumption during lockdown.

| Predictor | Coefficients β (*SE*) | *p-*value |
| --- | --- | --- |
| Outcome: Gaming |  |  |
| Intercept | ***-2.578 (0.123)*** | ***< .001*** |
| Work confinement | ***-0.006 (0.001)*** | ***< .001*** |
| Sex | ***1.736 (0.370)*** | ***< .001*** |
| Work confinement * Sex | ***0.006 (0.002)*** | ***.006*** |
| Outcome: News |  |  |
| Intercept | ***-1.434 (0.286)*** | ***< .001*** |
| Work confinement | ***0.018 (0.005)*** | ***.001*** |
| Living situation | **0.650 (0.322)** | **.046** |
| Work confinement * Living situation | ***-0.015 (0.006)*** | ***.009*** |
| *Note.* Italic beta coefficients and *p*-values are significant at *p* < .1. Bold beta coefficients and *p*-values are significant at *p* < .05. Bold and italic beta coefficients and *p*-values are significant at *p* < .01. | | |

Table S6. Increased screen use behaviors during lockdown predicted by social confinement.

| Outcome | Coefficients β (*SE*) | *p-*value |
| --- | --- | --- |
| TV | **0.007 (0.003)** | **.019** |
| Social Media | 0.003 (0.003) | .222 |
| News | 0.001 (0.003) | .766 |
| Gaming | *0.003 (0.002)* | *.052* |
| Internet | -0.001 (0.003) | .806 |
| *Note.* Italic beta coefficients and *p*-values are significant at *p* < .1. Bold beta coefficients and *p*-values are significant at *p* < .05. Bold and italic beta coefficients and *p*-values are significant at *p* < .01. | | |

Table S7. Increased screen use behaviors during lockdown predicted by social confinement and controlled for sex.

| Predictor | Coefficients β (*SE*) | *p-*value |
| --- | --- | --- |
| Outcome: TV |  |  |
| Intercept | ***-0.535 (0.142)*** | ***< .001*** |
| Social confinement | **0.006 (0.003)** | **.031** |
| Sex | 0.518 (0.325) | .114 |
| Outcome: Social Media |  |  |
| Intercept | *-0.307 (0.157)* | *.053* |
| Social confinement | 0.002 (0.002) | .329 |
| Sex | 0.041 (0.395) | .917 |
| Outcome: News |  |  |
| Intercept | ***-0.937 (0.151)*** | ***< .001*** |
| Social confinement | 0.002 (0.003) | .435 |
| Sex | 0.331 (0.310) | .288 |
| Outcome: Gaming |  |  |
| Intercept | ***-2.575 (0.123)*** | ***< .001*** |
| Social confinement | 0.002 (0.001) | .280 |
| Sex | ***1.751 (0.372)*** | ***< .001*** |
| Outcome: Internet |  |  |
| Intercept | ***-1.313 (0.147)*** | ***< .001*** |
| Social confinement | 0.002 (0.003) | .565 |
| Sex | *0.667 (0.358)* | *.065* |
| *Note.* Italic beta coefficients and *p*-values are significant at *p* < .1. Bold beta coefficients and *p*-values are significant at *p* < .05. Bold and italic beta coefficients and *p*-values are significant at *p* < .01. | | |

Table S8. Increased screen use behaviors during lockdown predicted by social confinement and controlled for age.

| Predictor | Coefficients β (*SE*) | *p-*value |
| --- | --- | --- |
| Outcome: TV |  |  |
| Intercept | ***-0.435 (0.129)*** | ***.001*** |
| Social confinement | **0.006 (0.003)** | **.033** |
| Age | 0.000 (0.016) | .976 |
| Outcome: Social Media |  |  |
| Intercept | **-0.301 (0.146)** | **.042** |
| Social confinement | 0.002 (0.002) | .330 |
| Age | **-0.027 (0.012)** | **.034** |
| Outcome: News |  |  |
| Intercept | ***-0.890 (0.130)*** | ***< .001*** |
| Social confinement | 0.002 (0.003) | .524 |
| Age | **0.038 (0.015)** | **.010** |
| Outcome: Gaming |  |  |
| Intercept | ***-2.046 (0.131)*** | ***< .001*** |
| Social confinement | ***0.003 (0.001)*** | ***.007*** |
| Age | *-0.013 (0.007)* | *.076* |
| Outcome: Internet |  |  |
| Intercept | ***-1.179 (0.135)*** | ***< .001*** |
| Social confinement | 0.002 (0.003) | .570 |
| Age | 0.015 (0.015) | .321 |
| *Note.* Italic beta coefficients and *p*-values are significant at *p* < .1. Bold beta coefficients and *p*-values are significant at *p* < .05. Bold and italic beta coefficients and *p*-values are significant at *p* < .01. | | |

Table S9. Increased screen use behaviors during lockdown predicted by social confinement and controlled for living situation (living alone or together with others).

| Predictor | Coefficients β (*SE*) | *p-*value |
| --- | --- | --- |
| Outcome: TV |  |  |
| Intercept | **-0.752 (0.375)** | **.048** |
| Social confinement | **0.006 (0.003)** | **.034** |
| Living situation | 0.374 (0.398) | .349 |
| Outcome: Social Media |  |  |
| Intercept | **-0.828 (0.347)** | **.019** |
| Social confinement | 0.002 (0.002) | .320 |
| Living situation | 0.626 (0.382) | .105 |
| Outcome: News |  |  |
| Intercept | ***-1.409 (0.271)*** | ***< .001*** |
| Social confinement | 0.002 (0.003) | .415 |
| Living situation | **0.627 (0.307)** | **.044** |
| Outcome: Gaming |  |  |
| Intercept | ***-1.872 (0.378)*** | ***< .001*** |
| Social confinement | ***0.003 (0.001)*** | ***.008*** |
| Living situation | -0.203 (0.402) | .615 |
| Outcome: Internet |  |  |
| Intercept | ***-1.087 (0.297)*** | ***< .001*** |
| Social confinement | 0.002 (0.003) | .575 |
| Living situation | -0.104 (0.335) | .757 |
| *Note.* Italic beta coefficients and *p*-values are significant at *p* < .1. Bold beta coefficients and *p*-values are significant at *p* < .05. Bold and italic beta coefficients and *p*-values are significant at *p* < .01. | | |

Table S10. Interaction effect of social confinement and age on increased television watching and increased social media usage during lockdown.

| Predictor | Coefficients β (*SE*) | *p-*value |
| --- | --- | --- |
| Outcome: TV |  |  |
| Intercept | ***-0.438 (0.129)*** | ***< .001*** |
| Social confinement | **0.006 (0.003)** | **.032** |
| Age | 0.006 (0.166) | .761 |
| Social confinement * Age | ***-0.001 (0.0002)*** | ***.004*** |
| Outcome: Social Media |  |  |
| Intercept | **-0.304 (0.146)** | **.039** |
| Social confinement | 0.002 (0.002) | .359 |
| Age | -0.008 (0.014) | .554 |
| Social confinement * Age | ***-0.001 (0.0002)*** | ***.001*** |
| *Note.* Italic beta coefficients and *p*-values are significant at *p* < .1. Bold beta coefficients and *p*-values are significant at *p* < .05. Bold and italic beta coefficients and *p*-values are significant at *p* < .01. | | |

Table S11. Increased screen use behaviors during lockdown predicted by social confinement and work confinement (controlling for shared variance).

| Predictor | Coefficients β (*SE*) | *p-*value |
| --- | --- | --- |
| Outcome: TV |  |  |
| Intercept | ***-0.433 (0.127)*** | ***< .001*** |
| Social confinement | **0.005 (0.003)** | **.039** |
| Work confinement | 0.001 (0.003) | .662 |
| Outcome: Social Media |  |  |
| Intercept | **-0.298 (0.145)** | **.042** |
| Social confinement | 0.002 (0.002 | .481 |
| Work confinement | **0.005 (0.002)** | **.048** |
| Outcome: News |  |  |
| Intercept | ***-0.856 (0.13)*** | ***< .001*** |
| Social confinement | 0.001 (0.03) | .592 |
| Work confinement | *0.004 (0.02)* | *.093* |
| Outcome: Gaming |  |  |
| Intercept | ***-1.84 (0.104)*** | ***< .001*** |
| Social confinement | ***0.004 (0.001)*** | ***< .001*** |
| Work confinement | ***-0.003 (0.001)*** | ***< .001*** |
| Outcome: Internet |  |  |
| Intercept | ***-1.132 (0.125)*** | ***< .001*** |
| Social confinement | 0.001 (0.003) | .584 |
| Work confinement | 0.001 (0.002) | .785 |
| *Note.* Italic beta coefficients and *p*-values are significant at *p* < .1. Bold beta coefficients and *p*-values are significant at *p* < .05. Bold and italic beta coefficients and *p*-values are significant at *p* < .01. | | |

Table S12. Increased screen use behaviors during lockdown predicted by day structure.

| Outcome | Coefficients β (*SE*) | *p-*value |
| --- | --- | --- |
| TV | ***-0.012 (0.003)*** | ***< .001*** |
| Social Media | 0.002 (0.002) | .406 |
| News | **0.005 (0.002)** | **.049** |
| Gaming | **-0.005 (0.002)** | **.014** |
| Internet | ***-0.006 (0.002)*** | ***.008*** |
| *Note.* Italic beta coefficients and *p*-values are significant at *p* < .1. Bold beta coefficients and *p*-values are significant at *p* < .05. Bold and italic beta coefficients and *p*-values are significant at *p* < .01. | | |
